# Skeleton-Based Activity Recognition for Children with Autism Using Graph Convolutional Networks

**DOI:** 10.3390/s26144638

**Published:** 2026-07-22

**Authors:** Betül Ay, Mehmet Ata Öztürk, Galip Aydın

**Affiliations:** 1 Department of Computer Engineering, Faculty of Engineering, Fırat University, 23119 Elazığ, Türkiye; gaydin@firat.edu.tr; 2 Department of Physical Education and Sports, Middle East Technical University, 06800 Ankara, Türkiye; mata@metu.edu.tr

**Keywords:** autism spectrum disorder, human activity recognition, 2D pose estimation, graph convolutional network, skeleton-based action recognition, deep learning, physical activity, privacy-preserving monitoring, video-based motion analysis

## Abstract

Movement-based and physical activity programs are central tools in autism intervention, so recognizing the activities a child performs during therapy is valuable for objective progress tracking. Manual monitoring of these sessions is time-consuming and subjective, and raw videos raise privacy concerns because it shows identifiable children. We address autism therapeutic activity recognition from privacy-preserving 2D skeletons, and we focus on the practical difficulty of how several therapeutic activities differ only in subtle motion details. As a backbone, we adopt ProtoGCN, a graph convolutional network that represents each action as a combination of learnable motion prototypes. However, this contrastive backbone organizes all classes at once, so it does not enforce a margin between the few pairs that remain entangled after training. We therefore introduce a Refine–Confusable (RC) module, a training-only regularizer that pushes apart the empirically most-confused class pairs using a hinge-margin loss over momentum-updated class centroids. The module changes neither the backbone nor the inference cost. On the MMASD dataset, restricted to the ten-class 2D-skeleton configuration, the RC module improves the base model across random, session-independent, and subject-independent evaluation. The gain is largest on the strictest subject-independent split and a clip-level analysis confirms that this improvement is statistically significant. Under the protocol-matched holdout, the method reaches 96.30% accuracy with 0.959 macro-F1, surpassing recent 2D-skeleton baselines while keeping a lightweight and privacy-preserving modality. The improvements are modest, as expected on a small clinical dataset, and t-SNE and prototype visualizations show that the learned representation is discriminative and interpretable.

## 1. Introduction

Activity recognition of autism-related behaviors in children has become a transformative tool for early screening, clinical diagnosis, and follow-up care [1,2]. Autism spectrum disorder (ASD) is a neurodevelopmental condition that affects social communication, behavior, and motor coordination from an early age [3]. Its presentation is heterogeneous, and the symptoms vary across age groups and between genders, which makes both diagnosis and progress tracking difficult [3,4]. Early and continuous intervention improves long-term outcomes, and it also helps families and educators respond to a child’s needs in a timely manner [4]. For these reasons, intervention sessions form a central part of autism care, and the value of each session depends on careful observation and assessment.

Movement-based and physical activity programs are among the most widely used intervention tools for children with autism. Structured play, rhythmic exercises, and adapted physical activity support motor development, and they also promote social engagement and self-regulation. Prior studies in adapted physical education report measurable benefits of such programs. For instance, a commercial exergaming intervention reduced stereotypical hand movements in children with ASD [5], while a game-console physical activity program improved physical fitness in individuals on the spectrum [6]. Elements drawn from structured teaching approaches have likewise been integrated into physical education for children with autism [7]. These findings show that the activities performed during a therapy session carry clinical meaning, so recognizing and quantifying them is useful for both therapists and researchers. Reviews of video-based motion analysis further report that motor atypicalities appear in a large majority of autistic individuals and can act as objective early markers, sometimes more informative than other behavioral signals [8].

The monitoring of intervention sessions is still largely manual. Therapists observe each child, record the activities performed, and judge the quality of movement, which is time-consuming and subjective [9]. As the number of children and sessions grows, manual analysis becomes a bottleneck, and differences between observers reduce the consistency of assessment. Automatic activity recognition addresses this gap because it labels the activities in a session without continuous human attention, and it provides an objective basis for progress tracking and individualized planning.

Two practical constraints shape the design of such a system. The first constraint is privacy, since therapy recordings show identifiable children in their homes and raw videos cannot be shared or processed freely [10]. A skeleton representation keeps the essential body motion while it removes appearance and identity, so it is a privacy-preserving alternative to raw frames [8,11]. Among skeleton modalities, the 2D skeleton is the lightest, because it stores only the x and y coordinates of the body joints. The second constraint is the fine-grained nature of therapeutic activities, since several activities differ only in subtle motion details. Two maracas-shaking variants, for example, differ only in the direction of shaking, and a model must separate these confusable pairs to produce a reliable session log.

Two 2D skeleton studies on MMASD define the starting point for our work, and each leaves a distinct gap. The first reaches a high headline accuracy, but it scores individual frames under a random split, so near-identical frames of one clip fall on both sides of the partition and the figure mainly reflects memorization [9]. The second adopts a leakage-aware session split and an honest protocol, yet it cannot separate a few visually similar activities such as singing and clapping against drumming, which the authors record as an open problem [12]. Our study is built around these two gaps. We report a rigorous leakage-free accuracy, and we add a mechanism that directly attacks the confusable-pair problem, while we keep the lightweight single-modality setting.

In this work we address autism therapeutic activity recognition from 2D skeletons, and we focus on the confusable-pair problem. We build on ProtoGCN, a graph convolutional network that represents each action as a combination of learnable motion prototypes and amplifies the subtle differences between similar actions [13]. ProtoGCN already includes a class-specific contrastive loss, yet this loss is a global objective that spreads its capacity over all classes and does not specifically guarantee a margin between the few pairs that remain entangled after training. We therefore propose a Refine–Confusable (RC) module, a lightweight regularizer that acts only on the empirically most-confused class pairs. The module reuses the momentum-updated class centroids of ProtoGCN, and it applies a targeted hinge-margin loss that pushes these specific clusters apart in the embedding space. The module is active only during training, so it introduces no architectural change and no extra cost at inference.

Our novelty is not a new backbone. It is the way we treat the residual confusions that a strong backbone leaves behind. A global contrastive loss organizes all classes at once and spreads its capacity uniformly, so it under-serves the few pairs that stay entangled after training. We instead identify these pairs from data and enforce a margin only between them at no inference cost. This reframes fine-grained activity recognition as a confusable-pair problem, and the mechanism transfers to other clinical movement tasks that face the same few-pair bottleneck, such as gait scoring or tremor typing.

We evaluate the method on the MMASD dataset of autism intervention recordings, restricted to the 2D-skeleton modality and to the ten-class configuration used by recent 2D studies [9,10,12]. The contributions of this paper are summarized as follows:

- We frame autism therapeutic activity recognition as a confusable-pair problem, and we show that misclassifications concentrate on a small set of similar activity pairs rather than spreading uniformly across classes.
- We propose the RC module, a training-only plug-in regularizer that enforces a margin between data-driven confusable pairs using momentum class centroids, without changing the backbone or the inference cost.
- We report a leakage-free evaluation with both random and subject-independent splits, and we observe gains in pooled accuracy under both, with the larger improvement on the stricter subject-independent setting. A per-fold analysis and a paired statistical test show that the effect is directionally consistent under all three protocols rather than tied to a single partition. The improvement is modest, it does not hold on every fold, and the paired test does not reach significance at five folds, so we read the per-fold results as supporting evidence rather than proof.
- We compare the method against recent 2D-skeleton baselines under a matched protocol and reach the highest reported accuracy, while we keep the privacy-preserving and lightweight 2D modality.
- We provide a qualitative analysis with t-SNE embeddings and a prototype usage map, which shows that the learned representation is discriminative and that the model assigns a dedicated subset of prototypes to each class.

**Figure 1:**
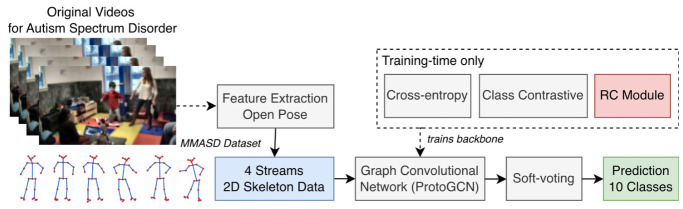
Overview of the RC-ProtoGCN pipeline.

Figure 1 gives an overview of the proposed RC-ProtoGCN pipeline. OpenPose extracts BODY_25 2D skeletons from the MMASD therapy videos, and we convert each clip into four streams of joint, bone, joint-motion, and bone-motion data. We train a separate ProtoGCN backbone for each stream, and we fuse the four softmax outputs by weighted soft-voting with weights of two, two, one, and one to predict one of ten activity classes. The cross-entropy loss, the class-specific contrastive loss, and the proposed RC module supervise the backbone only during training. The RC module, shown in red, is the contribution of this work, and it adds no cost at inference because it acts only on the training objective.

The rest of the paper is organized as follows. Section 2 reviews related datasets and methods. Section 3 summarizes the ProtoGCN backbone. Section 4 describes the proposed RC module. Section 5 presents the dataset, the preprocessing, and the evaluation protocol. Section 6 reports the results, Section 7 discusses them, and Section 8 concludes the paper.

## 2. Related Work

### 2.1. Datasets for Autism Activity Analysis

Three public datasets support most work on autism activity recognition. The Self-Stimulatory Behavior Dataset (SSBD) collects videos of stimming behaviors such as head banging and arm flapping that are gathered from public platforms [14]. The DREAM dataset records imitation and joint-attention tasks in robot-led and human-led sessions, and it provides skeleton data for movement analysis [15]. The MMASD dataset is the most recent and comprehensive of the three, and it targets play therapy for children with autism [10]. The cohort consists of thirty-two children, namely twenty-seven boys and five girls, aged between five and twelve years, who were recruited through local schools, services, and advocacy groups [10]. The recordings come from triadic play-therapy sessions in home settings, where a child, a therapist, and an adult model perform activities that target social and motor skills [10]. MMASD segments more than one hundred hours of therapy recordings into 1315 clips that cover eleven activities across three themes, namely robotic, music, and yoga. To preserve the privacy of the children, the dataset does not release raw video and instead provides four derived modalities, which are optical flow, 2D skeleton, 3D skeleton, and clinician evaluation scores. Because it is public, multimodal, and privacy-preserving, MMASD is well suited to a fair comparison between models, and we adopt it in this study. An enhanced version, MMASD+, was later released with 3D body mesh data and with person tracking that separates the therapist from the child [16]. Recent surveys of video-based and skeleton-based motion analysis for autism confirm the scarcity of public data in this area and list MMASD among the few public, multimodal, and privacy-preserving resources available [8,11].

### 2.2. Activity Recognition for Children with Autism

Two studies recognize MMASD activities directly from 2D skeletons, and both inform our design. Kanwal et al. proposed ARAIS, which feeds each frame as a vector of twenty-five joints into a stack of two LSTM layers and normalizes the coordinates by the frame dimensions [9]. The model reaches 95.72% accuracy, yet three properties inflate this figure. It classifies individual frames rather than clips, so a few hundred clips become hundreds of thousands of near-duplicate samples. It uses a random split that places frames of one child and even one clip on both sides of the partition, which lets the network memorize subject and clip appearance instead of the activity. It also divides the coordinates by the frame size, which keeps the absolute position in the scene, so the same action at the left or the right of the frame yields different inputs. Kassir et al. quantified the combined effect, since re-implementing ARAIS under a session split lowers its accuracy from 95.72% to 83.13%.

Kassir et al. addressed these issues with a leakage-aware and lightweight model [12]. They center each skeleton on the mid-hip joint and scale it by the shoulder width, which removes the dependence on position and body size, and we adopt the same normalization. They group frames by session, run a single eighty to twenty split with early stopping, and reach 94.84% accuracy with one modality, which approaches the multimodal MMASD+ result of 95.03% at a fraction of the cost. Their analysis leaves clear room for improvement. The model still confuses a few visually similar activities, most severely singing and clapping against drumming at 20.39%, maracas shaking against maracas forward shaking at 9.42%, and body swinging against chest expansion at 5.36%, and the authors state that the separation of similar activities needs further work. The input uses only raw joint coordinates, and the authors suggest adding joint velocities. The reported number also comes from a single split whose validation partition is reused for model selection, so its variance is unknown, and a bidirectional LSTM does not exploit the graph topology of the skeleton that helps separate localized motion differences.

A recent graph model approaches the same problem from the side of network topology rather than the training objective. Wang et al. proposed BERGCN, which reconstructs the spatial and temporal edges of the skeletal graph at the channel level from the differences between neighboring frames, so the graph adapts to the behavioral heterogeneity of each subject [2]. They evaluate the model on three autism tasks, including intervention activity recognition on MMASD, and, like us, they explore a multi-stream fusion over joint, velocity, bone, and bone-motion. On MMASD they report a Top-1 accuracy of 89.3%, yet this number is not directly comparable to ours, because they use the 3D skeleton with twenty-four joints, the eleven-class configuration, and a single random split, while we use the 2D skeleton, the ten-class configuration, and a leakage-free protocol. Their analysis still supports our motivation, since they report that activities from the same therapy scene are the most easily confused and they single out singing and clapping against drumming as a hard pair, which is the failure that we target. BERGCN handles this difficulty through the graph topology, while we handle it directly with a training-only objective that separates the specific pairs the base model confuses.

A separate line of work fuses several modalities. The MMASD+ extension adds a 3D body-mesh modality and combines a video vision transformer, a 3D convolutional network, and an LSTM with attention, which reaches 95.03% accuracy at a much higher computational cost [16]. Other studies on related datasets apply vision and vision–language models to stimming behaviors, and they obtain strong accuracy but depend on raw high-resolution video [17,18]. A recent dual-stream deep model instead analyzes 2D-skeleton body movement for autism and reports strong accuracy, which further supports the value of this lightweight modality [19]. A separate task on the same modality is the prediction of autism severity. Ranasingha et al. proposed a physics-informed tuple transformer that also operates on the 2D-skeleton stream of MMASD, yet it estimates the severity level of autism rather than the activity, and it takes the activity class as an input to the network [20]. Since that work recognizes severity while we recognize the activity, the two settings are complementary, and the reported accuracies are not directly comparable. Our work stays in the lightweight and privacy-preserving 2D-skeleton setting and answers these weaknesses by design rather than by adding modalities. We score at the clip level and evaluate with five-fold cross-validation under the random, session-independent, and subject-independent splits, which avoids the leakage of a frame-level random split and the optimism of a single reused holdout. We also replace the generic temporal model with a ProtoGCN backbone that exploits the graph topology of the skeleton, and we fuse a four-stream ensemble of joint, bone, joint-motion, and bone-motion so that motion cues such as joint velocities already enter the input. On top of this backbone, the RC module adds a targeted training objective for the confusable pairs that this prior work leaves unresolved, while the architecture and the inference cost stay unchanged.

### 2.3. Skeleton-Based Action Recognition

Skeleton-based action recognition models the human body as a graph of joints and bones, so graph convolutional networks are a natural fit. Early methods instead relied on handcrafted features, where joint positions were combined with joint velocities and the most motion-salient joints were selected to separate visually similar actions [21]. The spatial–temporal graph convolutional network introduced this paradigm and modeled the joint connections together with their motion over time [22]. Later models refined the adjacency structure, for example by learning channel-wise topologies that adapt the graph per feature channel [23]. ProtoGCN follows this family and observes that similar actions differ mainly in subtle local motion, so it represents each sample as an associative combination of learnable motion prototypes and amplifies the fine-grained differences [13]. To make these representations discriminative, ProtoGCN adds a class-specific contrastive loss that organizes the embedding space. Contrastive objectives of this kind are common in representation learning, and they typically use momentum-updated references and a margin or a temperature to control separation [24,25]. Recent surveys of pose-based autism analysis likewise note that graph-based and attention-based models are well suited to capturing the joint dependencies and long-range temporal structure of skeleton motion [8,11]. Graph and attention models remain the current choice for skeleton action recognition, and recent work continues to pair a graph-based skeleton branch with attention-based fusion for multimodal action recognition [26]. That work addresses general human activities, while our study targets the finer therapeutic activities of children with autism. Our RC module belongs to the same contrastive family, but it differs in scope, because it does not organize all classes at once and instead enforces a margin only between the specific pairs that the base model confuses.

### 2.4. Skeleton-Based Learning for Clinical Movement Analysis

Skeleton and pose data support movement analysis well beyond autism, and a broad clinical line motivates our design. In Parkinson’s disease, pose-based models estimate motor severity from video. Lu et al. extract 3D skeletons from monocular video and classify MDS-UPDRS gait scores [27]. Endo et al. pre-train a transformer on public motion data and transfer it to few-shot MDS-UPDRS gait estimation, which addresses the small size of clinical cohorts [28]. Zhang et al. classify Parkinsonian tremor from pose sequences with an interpretable graph network [29]. Related graph models target cerebral palsy risk from infant movement [30]. These works share our setting. They read fine-grained motion from privacy-preserving skeletons, they operate on small clinical datasets, and their residual errors concentrate on a few movement patterns that look alike. This is the same bottleneck that we attack. Our Refine–Confusable module is orthogonal to the backbone, since it is a training-only objective over class centroids, so it applies to any skeleton classifier in this family rather than to autism activity recognition alone.

## 3. Background: The ProtoGCN Architecture

Our method builds on ProtoGCN [13], so we first summarize its components and notation. The core idea of ProtoGCN is that similar actions differ only in subtle local motion, so the model represents each sample as a combination of learnable motion prototypes and amplifies the fine-grained differences. We keep the entire backbone unchanged and extend only its contrastive stage, which we revisit in Section 3.5.

### 3.1. Graph Convolution Preliminaries

The human skeleton is modeled as a graph with joints as nodes and bones as edges. The input is a feature tensor H ∈ ℝ^{N × T × C}, where N is the number of joints, T the number of frames, and C the number of channels. An adaptive graph convolutional network stacks L layers in total, and l indexes these layers. The transformation from layer l minus one to layer l is written as Equation (1),

(1) $$H^{\left(l\right)}=\sigma (A^{\left(l\right)}\;H^{\left(l-1\right)}\;W^{\left(l\right)})$$

where *H* is the layer feature tensor whose superscript marks the layer index, *A* is the adaptive graph topology of the layer, *W* is the learnable weight matrix, and the activation is the rectified linear unit. The final representation feeds a classifier that produces the class scores, and the network is supervised by the cross-entropy loss in Equation (2),

(2) $$L_{CE}=-\sum_{i}y_{i}\;log\;{ŷ}_{i}$$

where *y* is the ground-truth label and the predicted distribution is denoted with a hat.

### 3.2. Prototype Reconstruction Network

The prototype reconstruction network rebuilds the learned topology as a combination of stored prototypes. The adaptive topology is reshaped into a matrix X, and a learnable memory holds a set of prototype vectors, where each prototype encodes one relationship pattern between joints. A learnable query addresses the memory through attention and produces the response weights in Equation (3),

(3) $$R=softmax(X\;W_{query}^{T})$$

where *X* is the reshaped adaptive topology, the query matrix projects this input, and *R* collects the response weights that address the prototype memory. These weights linearly combine the prototypes to yield the enhanced fine-grained representation in Equation (4),

(4) $$Z=R\;\cdot \;W_{memory}$$

where the memory matrix stores the prototype vectors and *Z* is the enhanced fine-grained representation. This sample-and-assemble bottleneck forces every representation to be built only from discriminative prototypes, so it filters out non-discriminative noise.

### 3.3. Motion Topology Enhancement

Because the quality of *X* governs the prototype retrieval, the motion topology enhancement module enriches the topology at every layer. From the layer features the module computes query and key projections, and it models two complementary relations. The first relation captures intra-sample correlations through inner products, as shown in Equation (5),

(5) $$A_{intra}=\varphi (H^{Q}\;{\left(H^{K}\right)}^{T})$$

where the query and key projections are computed from the layer features and their inner product yields the intra-sample correlation map. The second relation captures inter-sample distinctions through pairwise joint differences, as shown in Equation (6),

(6) $$A_{inter}=\varphi (T_{1}\;H^{Q}-T_{2}\;H^{K})$$

where the query and key projections are first expanded into pairwise form over all joint pairs, and their difference yields the inter-sample distinction map. These enhanced topologies are added to the shared base topology and substituted into the graph convolution, which gives Equation (7),

(7) $$H^{\left(l\right)}=\sigma ((A_{0}+A_{intra}+A_{inter})\;H^{\left(l-1\right)}\;W^{\left(l\right)})$$

where the shared base topology and the two enhanced topologies are summed before the graph convolution, and the activation is the rectified linear unit.

### 3.4. Class-Specific Contrastive Learning

To make the reconstructed features discriminative, ProtoGCN adds a class-specific contrastive loss. The pooled feature is projected to an embedding *f*, and a memory bank holds one class aggregation per class. The aggregation of class *k* is updated, each batch with a momentum rule, as shown in Equation (8),

(8) $$m_{k}=\alpha \;m_{k}+(1-\alpha )\;{\bar{f}}_{k}$$

where the class mean is the in-batch mean of class *k* and the momentum is set to 0.9. The loss pulls each sample toward its own class aggregation and pushes it away from all other aggregations through a global softmax with temperature, as shown in Equation (9),

(9) $$L_{CSC}=-log\;\frac{exp(f\cdot m_{k}/\tau )}{exp(f\cdot m_{k}/\tau )+\sum_{l\neq k}exp(f\cdot m_{l}/\tau )}$$

where *f* is the sample embedding, the aggregation of its own class is the positive target, and the temperature scales the softmax over all class aggregations. The overall ProtoGCN objective combines the classification term and the contrastive term with a balance weight, as shown in Equation (10).

(10) $$L=L_{CE}+\lambda \;L_{CSC}$$

### 3.5. Connection to Our Method

The class-specific contrastive loss in Equation (9) is a global objective, since it organizes all classes at once through a softmax over every class aggregation. This design is effective in general, yet it distributes its capacity uniformly and does not specifically target the few class pairs that remain confusable after training. Our RC module keeps the entire ProtoGCN backbone and its contrastive loss intact, and it adds a focused margin-based regularizer that acts only on the empirically most-confused pairs. We reuse the idea of momentum-updated class centroids from Equation (8), but we employ them in a pairwise hinge-margin formulation rather than in a global softmax, so that we directly enforce a minimum separation between the specific clusters that the base model conflates.

## 4. Proposed Method: RC-ProtoGCN

### 4.1. Overview

We keep the entire ProtoGCN architecture of Section 3 unchanged, which includes the same backbone, the same prototype reconstruction network, the same motion topology enhancement, and the same class-specific contrastive loss. Our key observation is that, even with this contrastive loss, the misclassifications are not uniformly distributed and instead concentrate on a small set of confusable class pairs, for example maracas shaking against maracas forward shaking, or body swing against chest expansion. The reason is that the contrastive loss is a global objective whose softmax organizes all classes simultaneously and spreads its capacity uniformly, so it does not guarantee a margin between the few pairs that stay entangled after training. We therefore propose the Refine–Confusable module, which is a plug-in regularizer added on top of the ProtoGCN objective. The module reuses the momentum-updated class centroids, but instead of a global softmax, it applies a targeted hinge-margin loss that repels only the empirically most-confused class pairs. The module is active only during training, and it introduces no architectural change and no additional cost at inference.

### 4.2. Notation and Embedding Extraction

We deliberately reuse the symbols of Section 3. Let the full ProtoGCN backbone with its prototype reconstruction and topology enhancement be denoted by a single mapping. For an input clip, global average pooling of the backbone output gives the same embedding *f* that ProtoGCN feeds to its contrastive stage, as shown in Equation (11),

(11) $$f=GAP(\Phi (X))\;\in \;R^{d}$$

and the classifier produces the class scores over the action classes as in Section 3.1. For the pairwise distances we use the L2-normalized embedding in Equation (12).

(12) $$\tilde{f}\;=\;f/{\left\|f\right\|}_{2}$$

Normalization removes scale effects and renders the distances meaningful on the unit hypersphere, so that a single margin threshold is interpreted consistently across all classes. The contrastive loss of ProtoGCN operates on the same embedding *f*, which is exactly why our regularizer can share its embedding space without any additional projection.

### 4.3. Exponential Moving Average Class Centroids

We adopt the same class aggregation as ProtoGCN. For each class *k*, we maintain a centroid that is updated, each mini-batch with a momentum of 0.9, where the class mean is the in-batch mean of the embeddings of class *k*, as shown in Equation (13).

(13) $$m_{k}=\alpha \;m_{k}+(1-\alpha )\;{\bar{f}}_{k}$$

This update is identical in form to Equation (8), and the only difference is the use to which the centroid is put. The update is applied under a stop-gradient operation, so the centroid acts as a stable anchor. A single mini-batch yields a noisy centroid for a small class, whereas the momentum average provides a reliable estimate of each class cluster. While ProtoGCN consumes the centroid inside a global softmax, we consume the same centroid as a repulsion target in a pairwise hinge-margin, which we define next.

### 4.4. Confusable-Pair Contrastive Loss

Let the set of confusable class pairs be predefined and data-driven, where in our setup it holds eight pairs derived from the validation confusion matrix of the base ProtoGCN, as described in Section 4.6. For a mini-batch we collect the samples of each class, and we use the L2-normalized class centroid taken from Equation (13). With a margin set to 0.5 the loss consists of two terms. The first term is an in-batch class-mean margin, and it is active only when both classes of a pair appear in the batch. In that case we apply a margin to the distance between their mean embeddings, as shown in Equation (14),

(14) $$L_{mean}^{\left(a,b\right)}={\left[\rho -{\left\|{\tilde{f}}_{a}-{\tilde{f}}_{b}\right\|}_{2}\right]}_{+}$$

where the bracket with the plus sign denotes the hinge function that returns zero for negative arguments, and the class subscript denotes the mean of the normalized embeddings of that class. The second term is an instance-to-opposite-centroid repulsion, and it pushes each instance of one class away from the centroid of the other class, as shown in Equation (15).

(15) $$L_{ctr}^{\left(a,b\right)}=\frac{1}{\left|B_{a}\right|}\sum_{j\in B_{a}}{\left[\rho -{\left\|{\tilde{f}}_{j}-{\tilde{m}}_{b}\right\|}_{2}\right]}_{+}+\frac{1}{\left|B_{b}\right|}\sum_{j\in B_{b}}{\left[\rho -{\left\|{\tilde{f}}_{j}-{\tilde{m}}_{a}\right\|}_{2}\right]}_{+}$$

Because the centroids are detached, the gradients flow into the backbone only through the instance embeddings. The total pair loss is averaged over all pairs and over the active terms, as shown in Equation (16),

(16) $$L_{pair}=\frac{1}{N_{act}}\sum_{(a,b)\in P}\left(L_{mean}^{\left(a,b\right)}+L_{ctr}^{\left(a,b\right)}\right)$$

where the active count is the number of terms actually computed in that batch, which keeps the scale of the loss invariant to the batch composition. The two terms are complementary. The class-mean term gives an immediate and local separation signal, but it is not guaranteed at every step. The centroid term remains active even when only one class of the pair is present in the batch, because it repels against a global and persistent reference. In our data the target pairs co-occur in the same batch in about 91 percent of training batches, and the centroid term covers the one-sided case, so together the two terms keep the separation signal active at nearly every step and force the confusable clusters to stay at least a margin apart.

### 4.5. Overall Objective

We do not replace any part of the ProtoGCN objective. We simply add a third term to the existing cross-entropy and class-specific contrastive losses, as shown in Equation (17).

(17) $$L=L_{CE}+\lambda \;L_{CSC}+\gamma \;L_{pair}$$

The first two terms are exactly the ProtoGCN objective with its original weight, and the third term with weight *γ* is the only addition introduced by our method. We set the weight to 0.5 in all experiments.

### 4.6. Selection of Confusable Pairs

The set of confusable pairs is data-driven rather than hand-picked. We first train the base ProtoGCN under the random split and read its pooled out-of-fold confusion matrix, then we rank the class pairs by their bidirectional confusion, where the confusion of a pair is the number of clips of one class predicted as the other in either direction, and we keep the eight most-confused pairs as a fixed target set. Of the 45 possible class pairs, these eight account for 79 of the 134 base errors on the session-independent split, so a small fraction of the pairs carries the majority of the confusions while the remaining errors are spread thinly over many rare pairs. This procedure ties the regularizer to the errors that the base model actually makes, so the added pressure is spent where it is needed. The target set is chosen once, before the RC training, and it is not re-derived inside the cross-validation loop. Because the confusion matrix is a pooled out-of-fold estimate, each clip is predicted only when it is held out, so no clip contributes to the selection through its own training labels. The same fixed set is used for the session-independent and subject-independent evaluations, so the target pairs are never derived from the evaluation folds of those protocols and the selection cannot leak information from the test data. To confirm that the choice is stable rather than tied to a single fold, we repeat the ranking under a leave-one-fold-out scheme, where the eight pairs are selected from four folds at a time. The leave-one-fold-out sets recover the eight pairs with an average overlap of 6.8 of 8, and two of the five folds reproduce the full set exactly. The fixed set also transfers across protocols, since the eight pairs cover a comparable share of the base errors on the other splits, 43 of the 60 on the random split and 100 of the 149 on the subject-independent split. The most prominent pairs in our data are maracas shaking against maracas forward shaking and singing and clapping against drumming, which is consistent with the visual similarity of these activities.

### 4.7. Relation to Supervised Metric-Learning Losses

Our confusable-pair loss belongs to the family of supervised metric-learning objectives, so we clarify how it differs from common alternatives. Center loss pulls each sample toward its own class center and improves compactness, yet it adds no repulsion between specific classes, so it cannot open a margin on a chosen pair. Triplet loss operates on anchor, positive, and negative samples and needs careful triplet mining, which is costly and unstable on a small dataset. We avoid this mining, because we repel against momentum class centroids that give a stable reference at every step. ArcFace and Circle loss inject an angular or pairwise margin into the classification head, and this margin is global and uniform over all classes. Supervised contrastive learning is also global, since it pulls each sample to its own class and pushes it from every other class at once. The class-specific contrastive loss of ProtoGCN already plays this global role in our backbone. Our contribution is orthogonal to these global objectives. We keep the global loss and add a targeted term that spends its margin only on the few pairs the base model actually confuses. The term is training-only, it reuses the existing centroids, it changes neither the classifier nor the inference cost, and it needs no pair or triplet sampling. This focus is the reason we prefer it for the confusable-pair problem, where a small number of pairs carry most of the error.

## 5. Materials and Methods

### 5.1. Dataset and Activity Labels

We use the MMASD dataset of children with autism performing therapeutic activities, and we restrict it to the 2D-skeleton modality [10]. Each frame is one OpenPose (schema version 1.3) file in BODY_25 format [31], and every detected person carries a vector of twenty-five joints with x, y, and a confidence value. The activity label of a clip is taken from its folder name. Following both prior 2D works, we adopt the ten-class set that excludes frog pose, which allows a number-to-number comparison [9,12]. This configuration yields 1202 clips from 32 subjects. Table 1 lists the activities together with the number of segmented video clips per class. Each clip is represented as a sequence of sixty-four frames and classified at the clip level, and all aggregate metrics use macro averaging.

Our primary metric is a leakage-free five-fold cross-validation, and we report it both as the mean and standard deviation over the folds and as the pooled out-of-fold score over all clips. We use three splitting regimes. The first regime is a random split. The second regime is a session-independent split, in which all clips of one recording session are confined to a single fold, so no session is shared between training and test. The third regime is a stricter subject-independent split, in which all clips of one child are confined to a single fold, so no child appears in both training and test. To enable a direct comparison with prior work that uses a single train and test split, we additionally report a single eighty to twenty holdout with early stopping. The four streams that we describe in Section 5.2 are trained independently and fused by weighted soft-voting, with weights of two, two, one, and one.

### 5.2. Data Representation and Preprocessing

A filename in the release encodes the fields that we need for grouping and for leakage-free splitting. The clip key that groups frames into a sequence is the prefix up to the frame index, and the subject identifier enables the subject-independent split, and the subject and session together enable the session-independent split.

The bone and bone-motion streams operate on a fixed skeleton graph over the 25 BODY_25 joints. We connect the joints with 24 directed edges, where each edge runs from a child joint to its parent, and we compute the bone vector as the child position minus the parent position. The neck is the root and has no parent, so the 25 joints yield 24 bones. Table 2 lists the edges together with the body region that each one covers.

We convert the raw frames of each clip into fixed-length skeleton tensors through four steps. The first step is person selection, because OpenPose detects several people per frame without tracking identity. For each frame we keep a single skeleton, and we score the candidates by the number of valid joints first, by the mean confidence second, and by the proximity of the torso centroid to the previously selected centroid third, which suppresses identity switches. The second step is relative normalization, where the coordinates are centered on the mid-hip joint and scaled by the shoulder width. Frames whose mid-hip or shoulders are missing, or whose shoulder width falls below a small threshold, are zeroed, and the normalized coordinates are clipped to a fixed range while missing joints stay at the origin and act as a natural mask. The third step computes the bone and motion streams, since from the joint topology we derive bone vectors as the difference between a child joint and its parent, and we derive motion as the first-order difference between consecutive frames for both joints and bones. This produces four streams, namely joint, bone, joint motion, and bone motion. The fourth step is temporal sampling, where each variable-length clip is uniformly resampled to a fixed length of sixty-four frames.

Each clip is finally stored as a four-stream tensor with two coordinate channels, sixty-four frames, twenty-five joints, and a single person. At training time the four streams are fed to four independently trained ProtoGCN models whose softmax outputs are fused by weighted averaging, with weights of two for the joint stream, two for the bone stream, one for the joint-motion stream, and one for the bone-motion stream. We follow the standard four-stream fusion practice and weight the joint and bone streams twice as much as the two motion streams [22,23]. The motion streams are first-order temporal differences and form weaker individual classifiers, so we keep their weight lower, and we fix all weights in advance rather than tuning them on the evaluation data.

### 5.3. Implementation Details and Training Environment

Each stream is trained with stochastic gradient descent and Nesterov momentum of 0.9, an initial learning rate of 0.05, and a weight decay of 0.0005. The learning rate follows a multi-step schedule that scales it by 0.1 at seventy percent and at ninety percent of the epoch budget, and the batch size is thirty-two. We train for fifty epochs in the five-fold cross-validation and for one hundred epochs in the single eighty to twenty holdout. The four streams, namely joint, bone, joint motion, and bone motion, are trained independently and fused by weighted soft-voting with weights of two, two, one, and one. The objective combines the cross-entropy loss, the class-specific contrastive loss, and the RC pair-contrastive loss, where the RC module uses the data-driven set of eight confusable pairs, a pair weight of 0.5, a margin of 0.5, and a centroid momentum of 0.9. The RC term is active only during training, so the inference cost is identical to the base ProtoGCN. We train the base ProtoGCN with this same four-stream pipeline, protocol, and hyperparameters on the MMASD 2D skeletons, so the base and the RC model differ only in the added pair-contrastive term.

All models were trained on a single cloud node with one NVIDIA H100 80 GB graphics processing unit. Table 3 summarizes the hardware and software of the training environment. The RC module adds negligible overhead, because it only augments the loss and does not change the network.

### 5.4. Evaluation Protocol

In order to avoid the per-frame leakage that affects frame-level protocols, we score one label per clip, since the frames within a clip are near-duplicates. We evaluate every configuration with the leakage-free five-fold cross-validation described in Section 5.1, under the random, session-independent, and subject-independent splits. We report the cross-validation accuracy and the macro-F1 as the mean and standard deviation over the folds, and we also report the pooled out-of-fold accuracy, precision, recall, and F1 over all clips. To check whether the difference between RC-ProtoGCN and the base model is systematic, we also run a paired Wilcoxon signed-rank test on the per-fold accuracy differences and report the fraction of folds on which RC-ProtoGCN wins. A low variance across the folds indicates consistent generalization that does not depend on a single train–test split, which is an essential requirement for clinical use [32]. To compare with prior work that uses a single partition, we add a single eighty to twenty holdout with early stopping. We treat the five-fold cross-validation as the principal and leakage-free result, and we treat the holdout only as a protocol-matched point of comparison.

## 6. Results

We conduct several sets of experiments to demonstrate the effect of the RC module. In the first set of experiments, we compare RC-ProtoGCN against the base ProtoGCN under the leakage-free five-fold cross-validation. In the second set of experiments, we benchmark our method against recent 2D-skeleton work under a matched protocol. We then analyze the effect of the module on the confusable pairs, and we close with a qualitative analysis of the predictions. We present performance results for all configurations using a single NVIDIA H100 graphics processing unit.

### 6.1. Main Results: RC Module Against the Base ProtoGCN

Table 4 shows the experimental results of the five-fold cross-validation. The cross-validation columns report the mean and standard deviation over folds, while the pooled column reports the out-of-fold accuracy over all clips. The RC module improves the base ProtoGCN under all three splitting regimes. On the random split the cross-validation accuracy rises from 94.99% to 95.31%, and the pooled accuracy rises from 94.99% to 95.30%. On the session-independent split the cross-validation accuracy rises from 88.75% to 90.33%, and the pooled accuracy rises from 88.82% to 90.03%. On the stricter subject-independent split the cross-validation accuracy rises from 87.77% to 89.36%, and the pooled accuracy rises from 87.73% to 89.29%.

An interesting observation from Table 4 is that the gain is largest on the subject-independent split, which indicates that the targeted pair regularization is especially helpful when the model must generalize to unseen children, the setting that matters most for clinical deployment. On the subject-independent split, the RC module improves both the pooled accuracy and the macro-F1, and it also lowers the fold-to-fold variance. On the session-independent split, the pooled accuracy improves while the per-fold macro-F1 stays close to the base, because these folds are smaller and more heterogeneous. We also compare against two strong skeleton-based baselines, InfoGCN [33] and CTR-GCN [23], re-trained on MMASD under the same protocol. All models use the same four-stream inputs, the same five-fold splits and seed, and the same optimizer, schedule, and augmentation, so the comparison is controlled. The baselines differ from the base ProtoGCN only in the backbone, and RC-ProtoGCN differs from it only in the added training-only RC term. RC-ProtoGCN outperforms both on all three splits, and CTR-GCN is the strongest baseline since it exceeds the base ProtoGCN on all three splits.

Table 4 also reports the pooled macro-averaged precision, recall, and F1 for every configuration, together with the protocol-matched holdout. In Table 4, the CV columns report the mean and standard deviation over the five folds, and the remaining columns report the pooled out-of-fold macro-averaged metrics. The five-fold rows are pooled out-of-fold scores. The RC module improves all four pooled metrics over the base ProtoGCN under all three splits, and the best configuration reaches 96.30% accuracy with 0.959 macro-F1 on the holdout. The 80/20 holdout row reports a single test measurement, not cross-validation statistics; it is included only for comparison with ARAIS and Kassir et al. and should be read as an optimistic estimate.

**Figure 2:**
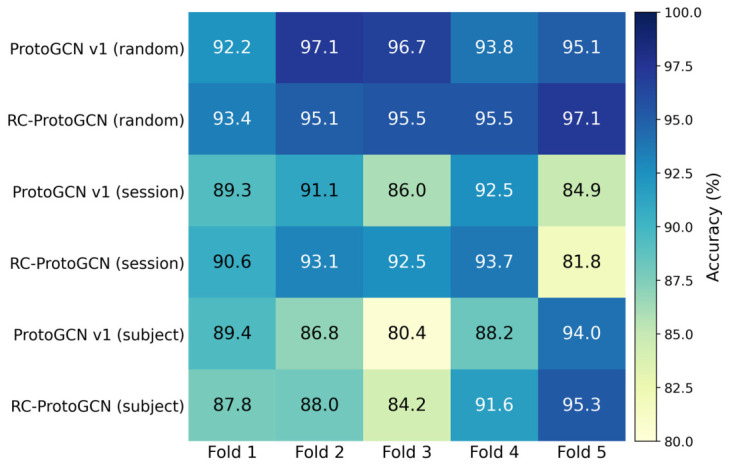
Fold-wise accuracy of the base ProtoGCN and RC-ProtoGCN under the random, session-independent, and subject-independent splits.

Figure 2 reports the accuracy of each model on each fold, where a darker cell marks a higher accuracy. The heatmap lets us check whether the mean accuracy is driven by a single favorable fold. RC-ProtoGCN on the random split stays uniformly high across all folds, since every fold reaches at least 93.4%, so its advantage does not depend on a single fold. On the session-independent split, the fifth fold is the clear weak point, where RC-ProtoGCN reaches 81.8% and the base model reaches 84.9%, because the subject and session combination of that fold is the hardest generalization case.

To test whether the improvement is systematic, we run a paired Wilcoxon signed-rank test on the per-fold accuracy differences between RC-ProtoGCN and the base model, and we also report the fraction of folds on which RC-ProtoGCN wins. RC-ProtoGCN wins on three of the five random folds, on four of the five session-independent folds, and on four of the five subject-independent folds. The paired Wilcoxon test returns *p*-values of 1.000, 0.4375, and 0.3125 on the random, session-independent, and subject-independent splits, respectively.

The Wilcoxon *p*-values do not fall below 0.05, yet this is a property of the fold count and not evidence against the method. With only five folds the smallest *p*-value that the test can return is 0.0625, so a value below 0.05 is mathematically impossible at this sample size. The substantive evidence is therefore the direction of the effect, which favors RC-ProtoGCN under all protocols and is strongest on the harder session-independent and subject-independent splits.

Because the fold-level test is limited by the small number of folds, we complement it with a clip-level analysis on the pooled out-of-fold predictions. For each protocol we compute a paired bootstrap 95 percent confidence interval for the difference in pooled accuracy between RC-ProtoGCN and the base model over ten thousand resamples, the paired effect size as Cohen’s d over the five folds, and a McNemar test on the clip-level agreement. Table 5 reports the result. On the strictest subject-independent split, the improvement is significant, since the confidence interval excludes zero, from 0.25 to 2.97 points, and the McNemar test gives a *p*-value of 0.032. On the session-independent split the interval is almost entirely positive and the effect size is moderate, while on the random split, the base model is already near the ceiling and the difference is not separable from zero. The clip-level analysis therefore supplies the stronger evidence that the fold-level test cannot reach at five folds, and it confirms that the gain is real and largest where generalization is hardest. Because Cohen’s d here is computed over only five folds, it is a noisy point estimate and should be read as an approximate indication of effect size rather than a precise value. The clip-level bootstrap interval and the McNemar test, which use far more samples, are therefore the primary evidence of significance.

### 6.2. Ablation Studies

We first isolate the two terms of the RC loss, the in-batch class-mean margin and the instance-to-centroid repulsion, and we train each one alone and both together. Table 6 reports the result. Accuracy is pooled out-of-fold, and the pair-error column counts bidirectional misclassifications on the eight target pairs under the session-independent split. On the random split all variants stay close to the strong base of 94.99 percent, and the full loss is best at 95.30 percent. On the harder session-independent split the full loss reaches the highest pooled accuracy at 90.03 percent, above the centroid term alone at 89.64 percent and the class-mean term alone at 89.29 percent.

The same ordering holds for the objective that the module targets, which is the number of bidirectional errors on the eight confusable pairs. The full loss is best and lowers these errors from 79 to 61, while the centroid term alone reaches 69 and the class-mean term alone reaches 71. We therefore keep both terms. The centroid term supplies a persistent signal through the momentum centroids, the class-mean term adds a batch-local margin when both classes are present, and together they give the largest reduction in the confusable-pair errors that motivate the module as well as the highest pooled accuracy on the harder split.

To check that the two terms are complementary rather than redundant, we simulate five thousand random training batches and count how often each term can fire on the target pairs. The class-mean term is active in 91.1 percent of pair-steps, since it needs both classes of a pair in the same batch, while the centroid term can contribute in 99.9 percent, since it needs only one class together with the momentum centroid of the other. At least one target pair appears in every batch. The remaining gap of about nine percent is exactly the set of steps where only the centroid term provides a gradient, which supports keeping both terms.

We next vary the number of target pairs. We fix the target set at the eight most bidirectionally confused pairs from the base out-of-fold confusion analysis, and we evaluate nested sets of four, eight, and twelve pairs. Table 7 reports the result on the session-independent split. The four pairs at the smallest setting cover the maracas and body swing confusions but leave out the tree pose, squat, and sing-and-clap pairs that the base model also confuses, namely tree pose against squat, body swing against squat, singing and clapping against drumming, and squatting against drumming, all of which enter at eight pairs. Extending to twelve pairs adds four lower-ranked pairs, maracas shaking against squatting, arm swing against squat, arm swing against tree pose, and maracas forward shaking against tree pose, that each carry few base errors and add margin pressure with little signal, which also makes the out-of-fold selection less stable. The three settings reach 90.94, 90.03, and 89.46 percent pooled accuracy, and this spread stays within the fold standard deviation of about four points, so no single setting is statistically dominant. We keep eight pairs as a data-driven and surgical regularization budget rather than a tuned hyperparameter, since it covers the majority of the base confusions, 79 of the 134 base errors on the session-independent split, while staying stable across folds, where a leave-one-fold-out selection recovers the eight pairs with an average overlap of 6.8 of 8.

### 6.3. Training Convergence

**Figure 3:**
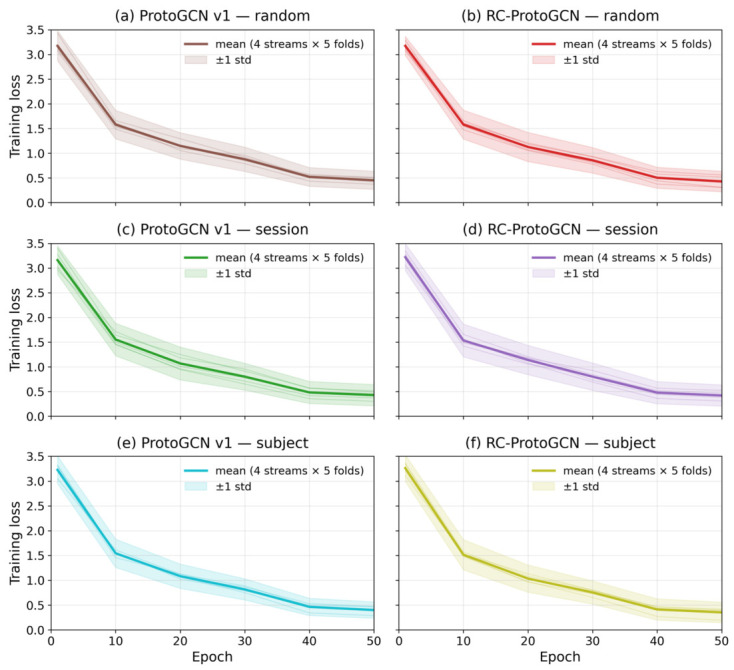
Training-loss curves for the base ProtoGCN and RC-ProtoGCN under the random, session-independent, and subject-independent splits.

Figure 3 shows the training-loss curves for the base ProtoGCN and for RC-ProtoGCN under the random, session-independent, and subject-independent splits, where each curve is the mean over the four streams and the five folds and the shaded band denotes one standard deviation. All six configurations converge smoothly and stably, and the added RC term does not destabilize the optimization. The RC model follows essentially the same convergence profile as the base model, which confirms that the accuracy gains come at no cost to the training stability.

**Figure 4:**
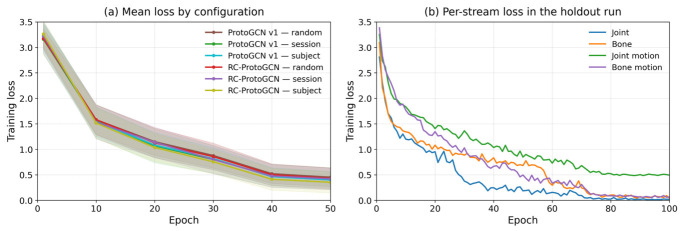
Training-loss analysis.

Figure 4 examines the convergence from two further angles. Panel (a) overlays the mean training loss of all six configurations on a single axis, and the curves nearly coincide, so the RC term tracks the base optimization closely and does not slow convergence. Panel (b) shows the per-stream training loss in the single eighty to twenty holdout over one hundred epochs for the joint, bone, joint-motion, and bone-motion streams. As we can observe from panel (b), the joint stream reaches the lowest loss, while the motion streams decay more slowly, which is consistent with the weighted fusion that gives a higher weight to the joint and bone streams. All streams reach a stable plateau, so each one contributes a well-trained classifier to the final soft-voting ensemble.

### 6.4. Comparison with Prior Work

Table 8 compares our method with recently reported results on MMASD 2D skeletons with ten classes. Kanwal et al. [9] uses a frame-level random split that is prone to leakage, while the results of [12] and ours are at the clip level. Under the single eighty to twenty holdout with early stopping, which is the protocol closest to [12], RC-ProtoGCN reaches 96.30% accuracy and 0.959 macro-F1. This surpasses both the bidirectional LSTM with multihead attention of [12] at 94.84% and the frame-level LSTM of the [9] study at 95.72%. The headline numbers in Table 8 understate the true gap, because the 95.72% of ARAIS is a frame-level score under a random split. When Kassir et al. re-implement ARAIS under a leakage-aware session split, its accuracy falls to 83.13% [12], so our method holds a much larger margin once the leakage is removed. Against this leakage-free 83.13%, RC-ProtoGCN reaches 90.03% under the five-fold session protocol. The two protocols are not identical, since the ARAIS figure comes from a single session split while ours is a five-fold cross-validation, yet both are leakage-aware, so the contrast shows that the near-parity of the headline numbers disappears once the frame-level leakage is removed. We emphasize that our principal and leakage-free metric remains the five-fold cross-validation result of 95.30% pooled accuracy on the random split. We provide the holdout row only for a protocol-matched comparison, and we note that it is mildly optimistic because the best epoch is selected on the same partition that is used for testing. We restrict Table 8 to methods that share our 2D, ten-class setting, so we do not list BERGCN [2] or the MMASD+ pipeline [16], because both rely on 3D inputs rather than the 2D skeleton.

### 6.5. Effect on Confusable Pairs

**Figure 5:**
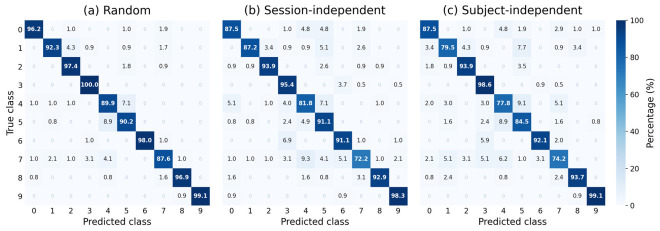
Confusion matrices of RC-ProtoGCN for (**a**) the random split, (**b**) the session-independent split, and (**c**) the subject-independent split.

**Figure 6:**
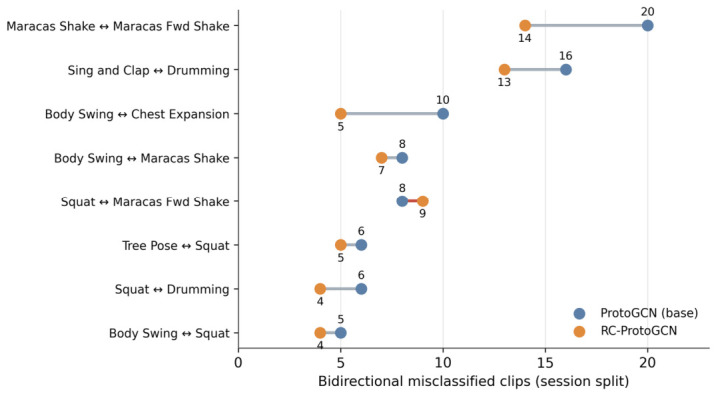
Per-pair bidirectional misclassified clips on the eight RC target pairs under the session-independent split, for the base ProtoGCN and RC-ProtoGCN.

Because the RC module specifically targets the most-confused class pairs, we examine its effect on those pairs directly. Figure 5 shows the confusion matrices of RC-ProtoGCN under the random split, the session-independent split, and the subject-independent split, where the values are row-normalized percentages. The class indices 0 to 9 follow the row order in Table 1. The diagonal stays strong in all three cases, and the largest off-diagonal mass concentrates on the maracas-shaking pair and on the sing-and-clap against drumming pair, which are exactly the visually similar activities. The improvement in the RC module is concentrated on these targeted pairs, while the already-separable classes are largely unaffected. This pattern validates the design goal of enforcing a minimum margin only between the clusters that the base model conflates. In aggregate, the RC module lowers the number of misclassified clips from 60 to 56 on the random protocol and from 134 to 120 on the session protocol. Figure 6 breaks the session reduction down by pair, where the bidirectional errors over the eight target pairs fall from 79 to 61. This reduction of eighteen clips exceeds the net session reduction of fourteen, so the gain is driven entirely by the targeted pairs while the few non-target classes show a small offsetting increase. In clinical terms, these eight pairs are distinct therapeutic activities that a session log must keep apart, so reducing their mutual confusion makes the recorded activity sequence more reliable for progress tracking.

### 6.6. Qualitative Analysis

The quantitative results show that the RC module raises accuracy and lowers the confusable-pair errors, but they do not reveal how the model behaves on individual clips or how it organizes the activities internally. We therefore complement the numbers with a qualitative study from three angles. We first inspect representative predictions to see which clips the model classifies correctly and which ones it still confuses. We then examine the learned feature space to check whether the confusable classes become separable after training. We finally look at the prototype usage to see whether the model assigns a dedicated set of prototypes to each activity.

**Figure 7:**
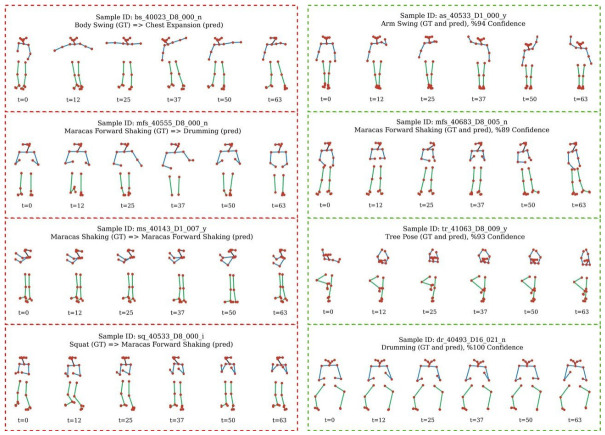
Representative skeleton sequences with ground-truth and predicted labels. Green marks correct predictions and red marks errors.

Figure 7 shows representative skeleton sequences with the ground-truth label and the predicted label. The correctly classified examples, drawn in green, cover both static poses and rhythmic actions, and the model assigns them high confidence. The misclassified examples, drawn in red, follow the confusable-pair pattern, since they involve activities with very similar upper-body motion such as the two maracas-shaking variants or body swing against chest expansion. These cases show that the remaining errors are driven by genuine motion similarity rather than by random noise, which is consistent with the confusion-matrix analysis and with the motivation for the RC module.

**Figure 8:**
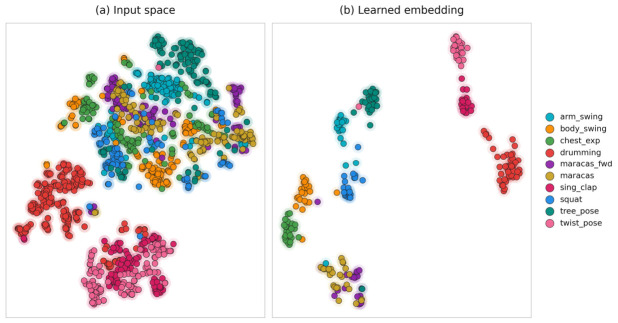
t-SNE visualization of the joint stream before and after learning.

Figure 8 projects the joint stream to two dimensions with t-SNE, both before and after learning. Figure 8a shows the raw joint-stream features of all clips before any training. The confusable classes overlap in this raw space and do not form separate groups, which motivates the need for a learned representation. Figure 8b shows the learned embedding of the test clips, where we take the 384-dimensional pooled features before the final classifier and project them with the same method. The classes of RC-ProtoGCN now form clean and well-separated clusters, which is direct evidence that the model learns discriminative representations. The contrast between the two panels confirms that the separation of the confusable activities comes from the learned embedding and not from the input features.

**Figure 9:**
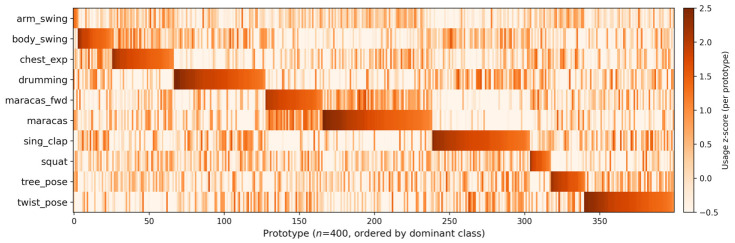
Prototype usage map of RC-ProtoGCN.

Figure 9 examines how RC-ProtoGCN uses its prototype bank. We capture the SoftMax attention over the 400 prototypes in the prototype reconstruction network of Section 3.2, and we record it for every graph edge. We average this attention over the edges to obtain the prototype usage of each clip, then we average over the clips of each class, and we apply a per-prototype z-score to enhance the contrast. When we order the prototypes by their dominant class, a clear block-diagonal structure appears, where each class strongly activates a dedicated subset of prototypes. This pattern shows that the model specializes its prototype bank in a class-specific way and that it learns interpretable and discrete action patterns.

This behavior follows from a stream-dependent allocation of capacity. The joint and joint-motion streams carry richer and more varied raw-coordinate configurations, so they receive a larger prototype bank. The bone and bone-motion streams are already relational, because each bone is a difference between two joints, so a more compact bank is sufficient for them. This design concentrates the capacity where it is most useful, and it limits the redundancy and the overfitting on the streams that need fewer prototypes.

### 6.7. Computational Cost

The RC module no trainable parameters and does not change the inference forward pass, since the pair-contrastive terms and the momentum centroid updates are applied only during training, and the centroids are maintained as non-parametric running statistics that are not part of the parameter budget. At inference the deployed model is exactly the base ProtoGCN, so the parameter count and the inference cost are identical to the baseline. Each stream has 4.33 million parameters and 1.93 GFLOPs per clip, and the four-stream ensemble has 16.84 million parameters and 7.72 GFLOPs. The only overhead is at training time. On an NVIDIA H100 80 GB GPU, enabling the RC loss increases the per-step training time from 48.8 to 55.0 milliseconds, about 12.7 percent, and the peak GPU memory from 4258 to 4281 megabytes, about 0.54 percent, measured over a short profiling run under the shared protocol. Table 9 reports these results.

## 7. Discussion

### 7.1. Interpretation of the Results

The results support the central idea of this work, namely that the recognition errors in autism therapeutic activities are not spread evenly across classes and instead concentrate on a few visually similar pairs. The base ProtoGCN already separates most activities well, and the RC module adds value precisely where the base model is weak. The gain is modest on the random split, where the test clips resemble the training clips, but it grows on the subject-independent split, where the model must recognize activities of unseen children. This behavior is encouraging for practice, because a monitoring tool will always face new children whose movement style differs from the training population.

The improvement is also stable rather than tied to a single fold. The per-fold heatmap shows that RC-ProtoGCN stays uniformly high across the random folds and wins on most folds under all protocols, so the gain reflects a consistent effect and not a favorable partition. A paired Wilcoxon test confirms the direction of this effect under all protocols, although its *p*-value cannot fall below 0.05 with only five folds, so we read the test as evidence of a consistent direction rather than as a significance threshold. This per-fold consistency matters for clinical use, because a tool that helps on average but fails on some sessions would be hard to trust.

The learned representation is also discriminative and interpretable, which supports the mechanism behind these gains. In the embedding space the confusable classes that overlap in the raw joint stream form clean and well-separated clusters, and the prototype usage map shows that each class activates a dedicated subset of prototypes. These two views indicate that the model does not only fit the labels but also organizes the motion patterns in a class-specific way, which is a desirable property when the output is meant to support a therapist.

On the large-scale NTU RGB+D and Kinetics benchmarks, ProtoGCN reports higher accuracy than CTR-GCN [13,23]. On the much smaller MMASD dataset used here, the base CTR-GCN instead slightly exceeds the base ProtoGCN on all three splits, which is consistent with the limited size of this clinical dataset, since the prototype-reconstruction backbone benefits from larger training sets. The ranking of the backbones is therefore dataset-dependent. Adding the RC term still places RC-ProtoGCN above CTR-GCN and every other baseline on all three splits, which indicates that the gain comes from the targeted pair regularization rather than from the choice of backbone.

### 7.2. Clinical Implications

The clinical motivation gives this improvement a concrete meaning. Physical activity and movement-based programs help children with autism develop motor and social skills, and prior work in adapted physical education reports reductions in stereotypical movement and gains in physical fitness after such programs [5,6]. Independent meta-analyses of randomized trials confirm that physical activity interventions improve motor and social outcomes in autistic children and adolescents [34]. A system that recognizes the activities of a session can support the therapist by producing an objective and consistent log of what the child performed, which complements the manual assessment and frees attention for direct interaction. This kind of objective and consistent monitoring fits the emphasis on careful evaluation and follow-up in pediatric autism care [35]. Automated movement analysis is also an active direction in this space. Two-dimensional pose estimation has been used to capture autism-relevant motor patterns in young children [36], and AI-based systems have advanced to prospective clinical evaluation [37]. The confusable-pair view is important here, because a session log is only useful if it separates similar activities reliably. A tool that confuses two maraca variants would record the wrong activity and could distort the progress record, so the targeted margin that the RC module enforces aligns directly with the needs of session monitoring.

Our design also respects the privacy constraints of this domain. We work entirely on 2D skeletons, which keep the body motion while they remove appearance and identity, and we never process raw video. The RC module preserves this property, since it changes only the training loss and leaves the lightweight 2D pipeline and the inference cost unchanged. The method therefore fits a setting where computational resources are limited and where the privacy of the children is a strict requirement.

### 7.3. Limitations and Future Work

Several limitations remain. First, the 2D skeleton discards depth, so two activities that differ mainly in the direction of motion can still project to similar 2D trajectories, which sets a natural ceiling on how far any 2D method can separate them [8]. Second, the skeletons are produced by a pose detector rather than by body-worn sensors, so detection failures under occlusion or poor lighting propagate into the recognition stage. Third, the confusable-pair set is fixed once it is derived from the base confusion matrix, so it does not adapt during training. Fourth, our evaluation uses one public dataset, and a broader study across sites and populations would strengthen the external validity, which is a constraint repeatedly highlighted across recent surveys of the field [8,11]. Fifth, the MMASD dataset is small, with 32 subjects and 1202 clips, so deep models can overfit and the improvements we report, on the order of one to one and a half points of pooled accuracy, should be read as consistent but modest gains rather than large effects. The training-only design of the RC module mitigates part of this risk, since the module adds no parameters at inference and does not increase the capacity of the deployed network, and the subject-independent protocol tests generalization to unseen children directly. Even so, the absolute numbers may not transfer to larger or more diverse clinical populations, and a study on additional cohorts would be needed to establish external validity.

These limitations point to clear directions for future work. Adding simple temporal cues such as joint velocities, or using a stronger pose estimator, could relieve part of the 2D ambiguity. A confusable-pair set that is refreshed during training could track the errors as they evolve. A multimodal extension that adds optical flow or 3D skeletons could be studied under the same privacy constraints, with attention to the extra cost [38,39]. Finally, a study with therapists and children in real sessions would test the practical value of the system, and a collaboration between computer engineering and adapted physical activity would help translate the recognition output into clinically meaningful feedback.

## 8. Conclusions

We presented RC-ProtoGCN, a skeleton-based method for recognizing the activities of children with autism during therapy. The method builds directly on ProtoGCN and adds a single targeted term to its objective. This term repels predetermined confusable class pairs through a two-part hinge-margin loss in the normalized embedding space, where the first part separates the in-batch class means and the second part pushes each instance away from the opposite class centroid. Unlike the global contrastive loss of ProtoGCN, which spreads its capacity over all classes, our term focuses only on the data-driven set of confusable pairs. The loss affects training alone, so the architecture and the inference cost stay identical to the base network. On the MMASD dataset with the ten-class 2D configuration, the RC module improves the base model under the random, session-independent, and subject-independent splits, with the largest gain on the subject-independent setting, and it reaches 96.30% accuracy with 0.959 macro-F1 under the protocol-matched holdout. We also presented detailed benchmarks against recent 2D-skeleton baselines, and we highlighted that the recognition errors concentrate on a small set of confusable pairs that the targeted term addresses. We further showed that the gain is directionally consistent under all three protocols, though modest and not uniform across every fold, and that the t-SNE and prototype visualizations make the learned representation discriminative and interpretable. The method keeps a lightweight and privacy-preserving 2D modality, which makes it suitable for objective monitoring of autism intervention sessions in resource-constrained and privacy-sensitive settings.

Several directions remain for future work, including real-time deployment during live therapy sessions, domain adaptation across clinics and populations, a multimodal extension that weights each modality by its reliability, and stronger clinical explainability with per-child personalization. Beyond its technical contribution, we hope this work also helps raise awareness of autism and shows how privacy-preserving and lightweight tools can support the children, the therapists, and the families who take part in intervention sessions.

## Figures and Tables

**Table 1:** Activity classes and clip counts in the ten-class MMASD 2D configuration [10].

Index	Activity Class	Description	Count
0	Arm swing	Raises the left and right arm one after the other while standing upright.	105
1	Body swing	Sways the body left and right with both arms stretched out, one trailing behind the other.	119
2	Chest expansion	Slowly opens and closes the chest.	114
3	Drumming	Plays a snare or Tubano drum with one or both hands.	168
4	Maracas forward shaking	Shakes maracas back and forth in front of the body.	103
5	Maracas shaking	Shakes maracas left and right in front of the chest.	130
6	Singing and clapping	Sits on the ground while singing and clapping at the same time.	113
7	Squat	Repeatedly lowers into a crouched stance with the knees bent.	101
8	Tree pose	Balances on one leg with the sole of the other foot against the standing leg.	129
9	Twist pose	Sits cross-legged and twists the torso to one side, keeping the lower body grounded.	120
—	Total	Ten activity classes across three themes (robotic, music, yoga)	1202

**Table 2:** Bone topology with the 24 directed edges used by the bone and bone-motion streams.

2D Skeleton Joints	Edge (idx)	Child Joint	Parent Joint	Body Part
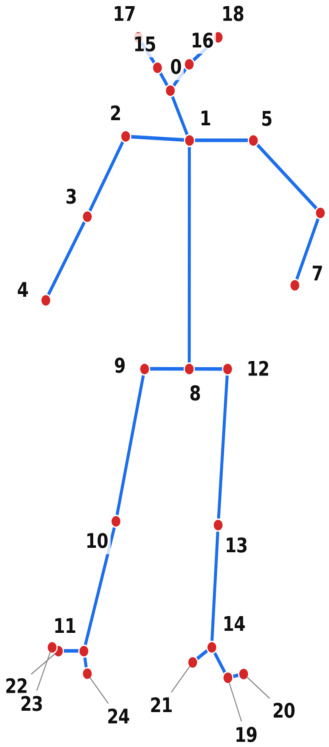	(1, 0)	Neck	Nose	Head/Trunk
	(1, 2)	Neck	RShoulder	Trunk
	(2, 3)	RShoulder	RElbow	Right arm
	(3, 4)	RElbow	RWrist	Right arm
	(1, 5)	Neck	LShoulder	Trunk
	(5, 6)	LShoulder	LElbow	Left arm
	(6, 7)	LElbow	LWrist	Left arm
	(1, 8)	Neck	MidHip	Trunk
	(8, 9)	MidHip	RHip	Right leg
	(9, 10)	RHip	RKnee	Right leg
	(10, 11)	RKnee	RAnkle	Right leg
	(8, 12)	MidHip	LHip	Left leg
	(12, 13)	LHip	LKnee	Left leg
	(13, 14)	LKnee	LAnkle	Left leg
	(0, 15)	Nose	REye	Head/Face
	(15, 17)	REye	REar	Head/Face
	(0, 16)	Nose	LEye	Head/Face
	(16, 18)	LEye	LEar	Head/Face
	(14, 19)	LAnkle	LBigToe	Left foot
	(19, 20)	LBigToe	LSmallToe	Left foot
	(14, 21)	LAnkle	LHeel	Left foot
	(11, 22)	RAnkle	RBigToe	Right foot
	(22, 23)	RBigToe	RSmallToe	Right foot
	(11, 24)	RAnkle	RHeel	Right foot

**Table 3:** Hardware and software of the training environment.

Component	Specification
GPU	NVIDIA H100 80 GB HBM3 (single device)
NVIDIA driver	570.211.01
CPU	224 vCPU
System memory	1.5 TiB
Operating system	Ubuntu 22.04.3 LTS
Python	3.10
Deep learning framework	PyTorch 2.1.0 (CUDA 11.8 build)
cuDNN	8.7.0

**Table 4:** Cross-validation and pooled results on MMASD 2D (ten classes).

Model	Protocol	CV Accuracy	CV Macro-F1	Accuracy	Precision	Recall	F1
ProtoGCN	random	94.99% ± 1.82%	94.59% ± 1.93%	94.99%	94.75%	94.49%	94.59%
RC-ProtoGCN	random	95.31% ± 1.18%	94.84% ± 1.33%	95.30%	95.01%	94.76%	94.86%
InfoGCN	random	94.73% ± 0.48%	94.40% ± 0.51%	94.73%	94.58%	94.32%	94.43%
CTR-GCN	random	95.06% ± 0.90%	94.66% ± 0.93%	95.06%	94.81%	94.62%	94.68%
ProtoGCN	session-independent	88.75% ± 2.91%	87.51% ± 2.82%	88.82%	88.52%	87.88%	88.09%
RC-ProtoGCN	session-independent	90.33% ± 4.40%	87.06% ± 4.83%	90.03%	89.79%	89.12%	89.33%
InfoGCN	session-independent	87.95% ± 4.37%	85.34% ± 5.40%	87.81%	87.67%	87.05%	87.17%
CTR-GCN	session-independent	89.95% ± 3.57%	88.77% ± 3.86%	89.82%	89.55%	89.03%	89.12%
ProtoGCN	subject-independent	87.77% ± 4.39%	86.58% ± 5.07%	87.73%	87.45%	86.79%	87.03%
RC-ProtoGCN	subject-independent	89.36% ± 3.78%	88.12% ± 4.24%	89.29%	88.75%	88.09%	88.37%
InfoGCN	subject-independent	86.77% ± 3.54%	85.77% ± 3.80%	86.74%	86.51%	85.57%	85.79%
CTR-GCN	subject-independent	88.51% ± 4.23%	87.70% ± 5.37%	88.46%	88.13%	87.82%	87.96%
RC-ProtoGCN	80/20 holdout	—	—	96.30%	96.44%	95.69%	95.94%

**Table 5:** Clip-level statistical comparison of RC-ProtoGCN and the base ProtoGCN on the pooled out-of-fold predictions.

Protocol	Δacc (pp)	95% CI (pp)	Cohen’s d	McNemar *p*
random	+0.31	[−0.74, 1.40]	0.31	0.644
session-independent	+1.21	[−0.08, 2.55]	0.504	0.096
subject-independent	+1.56	[0.25, 2.97]	0.738	0.032

**Table 6:** Component ablation of the RC loss on MMASD 2D (ten classes).

Variant	Random Acc.	Session Acc.	8-Pair Errors
ProtoGCN (base)	94.99%	88.82%	79
+ L_mean only	94.89%	89.29%	71
+ L_ctr only	95.14%	89.64%	69
RC-ProtoGCN (full)	95.30%	90.03%	61

**Table 7:** Effect of the number of target pairs on RC-ProtoGCN on the session-independent split.

|P|	Pooled Acc.	CV Acc.	Δ vs. |P| = 8
4	90.94%	91.27% ± 3.93%	+0.91
8 (reference)	90.03%	90.33% ± 4.40%	0.00
12	89.46%	89.92% ± 4.30%	−0.58

**Table 8:** Comparison with prior work on MMASD 2D skeletons.

Study	Model	Protocol	Accuracy	Macro-F1
ARAIS [9]	LSTM	80/20 holdout, early stop	95.72%	0.954
ARAIS re-impl. by [12]	LSTM	Session-based split	83.13%	-
Kassir et al. [12]	BiLSTM + attention	80/20 holdout, early stop	94.84%	0.940
RC-ProtoGCN (ours)	Prototype GCN + RC	80/20 holdout, early stop	96.30%	0.959
ProtoGCN [13]	Prototype GCN	5-fold pooled (random)	94.99%	0.946
InfoGCN [33]	GCN baseline	5-fold pooled (random)	94.73%	0.944
CTR-GCN [23]	GCN baseline	5-fold pooled (random)	95.06%	0.947
RC-ProtoGCN (ours)	Prototype GCN + RC	5-fold pooled (random)	95.30%	0.949
ProtoGCN [13]	Prototype GCN	5-fold pooled (session)	88.82%	0.881
InfoGCN [33]	GCN baseline	5-fold pooled (session)	87.81%	0.872
CTR-GCN [23]	GCN baseline	5-fold pooled (session)	89.82%	0.891
RC-ProtoGCN (ours)	Prototype GCN + RC	5-fold pooled (session)	90.03%	0.893
ProtoGCN [13]	Prototype GCN	5-fold pooled (subject)	87.73%	0.870
InfoGCN [33]	GCN baseline	5-fold pooled (subject)	86.74%	0.858
CTR-GCN [23]	GCN baseline	5-fold pooled (subject)	88.46%	0.880
RC-ProtoGCN (ours)	Prototype GCN + RC	5-fold pooled (subject)	89.29%	0.884

**Table 9:** Computational cost of the base ProtoGCN and RC-ProtoGCN.

Metric	Base ProtoGCN	RC-ProtoGCN	Δ
Trainable parameters (per stream)	4.33 M	4.33 M	0
Inference FLOPs per clip (per stream)	1.93 G	1.93 G	0%
Trainable parameters (four-stream)	16.84 M	16.84 M	0
Inference FLOPs per clip (four-stream)	7.72 G	7.72 G	0%
Training step time (batch 32)	48.8 ms	55.0 ms	+12.7%
Peak GPU memory	4258 MB	4281 MB	+0.54%

## Data Availability

The MMASD dataset is publicly available through the MMASD project website maintained by the original authors. The processing code developed for this study is available from the corresponding author on reasonable request.
